# Uterine Cancer

**Published:** 1889-12-07

**Authors:** 


### WITHIN THE WARDS.

### UTERINE CANCER.

There seems to be no doubt that cancer is increasing in this country, or, if it be not increasing, there is a larger proportion of cases brought to light than formerly. According to Dr. More Madden, obstetric physician to the Mater Misericordiæ Hospital of Dublin, the Registrar-General's statistics for the ten years ending 1887 show a decided addition to the number of certified deaths from cancer in proportion to the population. This, of course, does not, by itself, prove that there were more actual cases of the disease, but only that more were discovered by medical men. There is a presumption, of course, that the statistics represent an undoubted increase; but as it is certain that medical skill has greatly improved throughout the country, even during the last ten years, we may indulge the hope that the apparent increase is—partly, at any rate—the consequence of better diagnosis. Dr. Madden holds, and very justly, that the physician who undertakes to publicly discuss the treatment of cancer should be conscientiously accurate in his statement of facts. Only thus can real progress be made. The man who tries experiments insufficiently, and announces with a flourish of trumpets the results which he wishes he had achieved rather than those actually obtained, is infamous. Yet this has often been done, even by qualified and "honourable" medical men.

It cannot be fairly said that cancer is always and everywhere incurable. Dr. Madden agrees with Esmarch and other eminent surgeons and physicians that cases of undoubted cancer, even of the womb, the special form of cancer he has under consideration, may certainly be cured. But by what means is the cure to be effected? This is asked in order that the conviction may be plainly expressed that the question of means is comparatively unimportant. The real question is not one of methods, but of time. The patient should not ask, "How can any given cancer be cured?" But, "When can it be cured?" That is really the most important consideration in the whole business. The writer knows several ladies who had small tumours of the breast which were steadily growing larger. As soon as they were discovered, and it was made certain that they were actually growing, so soon was it resolved that they should be removed. He can call to mind, without his notebook, three ladies who are now living and well seven and eight years after operation, and one who is also quite well five years after. He can, unhappily, call to mind other cases which, because the patients were advised to wait and see what the growth would develop into, went all wrong within a year or two after operation, and ended in prolonged suffering and death. "When," therefore, "can cancer be cured?" is the vital question; and not "How?"

This is Dr. More Madden's view with regard to cancers of the uterus. In the great majority of the cases that have come under his notice the disease has commenced in the neck of the organ, and has remained localised there for a considerable period. The time for operation, then, is as soon as the discovery is certainly made that a growth of a doubtful nature is present at that spot. In many cases, when the neck has been removed quite early, the disease has been permanently arrested and the patient completely cured. Whereas, on the other hand, when time has been wasted in waiting to see whether the growth would extend or not, and whether it was really cancerous or not, the patient, though operated upon, and the whole uterus removed, has very much more frequently and hopelessly died. Dr. Madden, therefore, most strongly advises the immediate removal of the affected part of the organ the moment the discovery of an actual or possible cancer is made.

All married women will do well to note certain conclusions arrived at by such distinguished authorities as Mr. Jonathan Hutchinson and Dr. John Williams as to the origin and commencement of uterine cancer. These eminent scientists are of opinion that a large number of cancers of the womb have no other initial cause than lacerations caused by childbirth. Such lacerations may be perfectly harmless at the time, and may remain so; but if for any reason they take on inflammatory action, either at the period of parturition or thereafter, the inflammation may cause cell-growth and thickening of the tissues; it may spread by its inherent infectivity to contiguous parts, causing in them also cell-growth and thickening of the tissues. The new growth may remain quiescent for a time, and then may break down, causing more inflammation and more infection, and this very frequently results in fully-developed and fatal cancer of the womb. No doubt this sounds alarming. But, as a matter of fact, such alarm is the parent of safety, inasmuch as "to be forewarned is to be forearmed." The one thing for married and other women to bear in mind is this: at the first indication of internal inflammatory action competent medical aid is to be sought. Undoubtedly hundreds of lives which are now lost might be saved if everybody understood the necessity for curing all internal inflammatory attacks, however slight, at the very outset. Where one unmarried woman develops cancer of the neck of the womb ten married women suffer from the disease. It is clear, therefore, that all symptoms of pain or inflammation following either immediately or remotely after confinement should be considered as significant of approaching danger and should be got rid of without delay.

